# Correlates of Process of Suicide Attempt and Perception of Its Prevention

**Published:** 2016-07

**Authors:** Dushad Ram, Darshan Mahegowda, Basavana Gowdappa H

**Affiliations:** 1 Department of Psychiatry, JSS Medical College, Mysore, Karnakata, India. Email:akashji1972@gmail.com; 2 Department of Psychiatry, JSS Medical College, Mysore, Karnakata, India.‎ Email: msdarshan.mbbs@gmail.com; 3 Department of Medicine, JSS Medical College and Hospital, MG Road Agrahara, Mysore, Karnataka, India. Email: hbgowda@gmail.com

**Keywords:** *Correlation*, *Process of Suicide Attempt*, *Perception of Suicide Prevention*

## Abstract

**Objective: **Suicide attempt may follow a process right from the inception of the first information about ‎suicide until the act itself. This study was conducted to determine the relationship between ‎perception of suicide prevention with the process of suicide attempt and demographic ‎variables following a suicidal attempt.‎

**Method: **In this hospital based cross-sectional study, 168 consecutive admitted participants with a ‎suicide attempt were screened, and 109 who met the study criteria were recruited to ‎participate in this study before discharge. They were assessed using the socio-demographic ‎and clinical proforma designed for this study as well as by the Pierce Suicide Intent Scale. To ‎assess the process of suicide attempt and perception of suicide prevention, a 17- item ‎questionnaire was developed and used after rigorous literature search. The Cronbach’s ‎alpha coefficient value of this questionnaire found to be 0.84 in the reliability analysis.‎

**Results: **Media was the first source of information, and the majority had short duration of ‎preoccupation and interval between making the decision and the actual attempt and the ‎control of emotion during the attempt. A significant positive correlation was observed ‎between the source of the first information and age (p<01), reason for the method used and ‎economic status (p<01), duration since the first information and family history of suicide (p<01). Psychiatric diagnosis had a statistically significant association with the method used (p<01), duration of preoccupation (p<01), preparedness (p<01) and emotional state during ‎the attempt (p<01). A statistically significant negative correlation was found between the ‎source of the first information and education (p<01), any psychiatric diagnosis and duration ‎since the first death wish (p<01). On the score of perception about suicide prevention, a ‎significant group difference was observed for marital status, occupation, medical diagnosis, ‎opinion about an attempt, duration since the decision to attempt, and emotional control ‎during the attempt.‎

**Conclusion: **Based on the findings, it can be concluded that perception of suicide prevention may vary ‎with the process of suicide attempts and demographic characteristics.‎

Lifetime prevalence of suicide varies from 0.72 to 5.93% (1). It takes a form of process ‎‎ (process of suicide attempt), from an inception of the first information about suicide to an ‎actual attempt (2). It is a psychological process of self-destruction leading a person to death ‎‎ (3). There is dearth of studies examining the process of suicide attempt in the chronological ‎perspective. Few studies that adopted non-chronological approach (which focused on ‎duration of suicidal ideation and attempt) detected the following factors immediately after ‎a suicide attempt: Younger age at first death wish; fleeting suicidal thought months before ‎suicide; varied interval between communicating about suicide and actual act; perceived ‎suicide as a solution for problems and feeling emptiness and sadness (4,5 and 6). Reviews and ‎management guidelines on suicide attempt have emphasized‎ on interrupting the process of ‎suicide by providing appropriate education and awareness, proper media reporting, ‎screening and means restriction etc. (7). Understanding the relationship between the ‎process of suicide attempt and perception of suicide prevention may have therapeutic and ‎preventive implication. This study was conducted to determine the relationship between ‎perception of suicide prevention and the process of suicide attempt and demographic ‎variables following a suicidal attempt. We hypothesized that perception of suicide ‎prevention varies with the process of suicide attempt and demographic characteristics.‎ ‎

## Materials and Method

This cross-sectional, hospital-based study was conducted at JSS Hospital Mysore- Karnataka ‎‎ (India) after receiving the approval of institutional Ethical Committee. The study centre is a ‎non-profit organization catering affordable service to all socioeconomic classes and covers ‎more than about 10 lakhs population. All patients admitted for a suicide attempt from ‎September 2009 to June 2010 in Intensive Care Unit were screened and recruited after ‎recovery with treatment before discharge. Out of 168 patients screened, 109 met the ‎selection criteria, and were included in the study, and informed consent was obtained. ‎Inclusion criteria were patients of both genders, aged 16-65 years, history of severe suicide attempt ‎‎(suicidal attempt requiring significant medical treatment or causing severe injury that may lead to a death or ‎being associated with permanent medical squeal) and ≤ 15 days since the suicide attempt ‎‎(for better recall accuracy). Due to the reliability issues, participants were excluded if they ‎had an ICD 10 diagnosis of dementia or mental retardation, presence of psychotic symptoms ‎or any condition associated with significant impaired cognitive function during the attempt ‎or if they were unable to provide reliable information about their suicidal behavior. ‎Participants were excluded if they were on treatment for any psychiatric disorder, suffered ‎from any severe physical illness, or terminal physical illness as they may directly influence ‎the perception of suicide prevention. The study did not interfere with the ongoing treatment ‎by the physicians. All included patients were assessed with socio-demographic and clinical ‎Proforma designed for this study, Questionnaire of Process of Suicide Attempt and Pierce ‎Suicide Intent Scale (PSIS) (8). To assess the process of suicide attempt and perception of ‎suicide prevention, a 20- item questionnaire was developed after rigorous literature search. ‎Spearman correlation test was applied to evaluate the validity of the score on Pierce Suicide ‎Intent Scale. The underlying hypothesis was as follows: The score on the process of suicide ‎attempt questionnaire will correlate with the score on Pierce Suicide Intent Scale. A pilot ‎study was conducted on 20 participants. Three items did not have a statistically significant ‎correlation, so they were removed; and 17 items were used in this study. Each item had ‎three possible responses. The questionnaire assessed the initial reception of information ‎about suicide (Items 1, 2), activation of suicidal thoughts/ideas (Items 3-5), intense ‎suicidal state (Items 6-16) and post attempt state (Item 17). Reliability analysis revealed a ‎Cronbach’s alpha coefficient value of 0.84. A qualified psychiatrist performed all the ‎assessments. After the assessment, a brief psychological intervention (Life Skill Training) ‎was conducted with a focus on problem solving, effective communication, interpersonal ‎relationship, and coping with stress and emotion. An appropriate psychopharmacotherapy ‎was started depending on the psychiatric diagnosis after evaluation.‎

Statistical analysis was done using SPSS 16. Descriptive data were expressed with frequency, ‎percentages; and statistical significance, fixed at .05 and .01 level, was denoted with p ‎values.‎

Bivariate correlations procedure (Pearson’s correlation coefficient) was used to measure ‎the linear association between the variables. Because each item in the questionnaire of the ‎process of suicide attempt measures different dimensions, their correlation with ‎demographic variables were assessed. Similar correlation was assessed for the total score ‎on PSIS and variables of process of suicide attempt. As the dependent variable was ‎categorical, ANOVA test was carried out to determine the group difference in the ‎perception of suicide prevention vs. variables of socio-demography and questionnaire for ‎the process of suicide attempt.‎

## Results

The process of suicide attempts were characterized as follows: More participants with a ‎long duration since the first information about suicide, media as the first source of ‎information, short duration of interval from decision to attempt, preoccupation, and not ‎being prepared for an attempt. During the attempt, they felt some satisfaction for the act, ‎and were little sad but were in control of their emotion (Table 1).‎

Table 2 reveals the correlation of process of suicide attempt with demographic and clinical variables. A positive correlation was ‎observed between age and source of the first information (<0.01), economic status and ‎reason for the method used (<0.01), family history of suicide and duration of the first ‎information (<0.01). A negative correlation was observed between education and source of ‎the first information (<0.01), any psychiatric diagnosis and duration since the first death wish ‎‎ (<0.01). Any psychiatric diagnosis was positively correlated with the duration of decision to ‎attempt (<0.01), duration of preoccupation (<0.01), preparedness (<0.01) and emotional state ‎during the attempt (<0.01). No statistically significant correlation was observed for the score ‎on the Process of Suicide Attempt Questionnaire and demographic variables. PSIS score had ‎a positive correlation with most variables of the suicide process.‎

Table 3 displays only the statistically significant group difference on the score of suicide ‎prevention and variables of demographic characteristics and process of suicide attempt. On ‎the scores of perception about suicide prevention, a significant group difference was ‎observed for occupation, medical diagnosis, prevention of others from suicide and opinion ‎about an attempt.‎

**Table1:** Characteristics of the Process of Suicidal Attempt

**Variables**		**N**	**%**
**Duration since first information about suicide**	Months -years back Weeks back Few days back	104 1 4	95.4 0.9 3.7
**Source of first information**	Through media Through society Through family member, near or dear	77 27 5	70.6 24.8 4.6
**Duration since first death wish**	Months -years back Weeks back This was the first time	35 19 55	32.1 17.4 50.5
**Duration of decision to attempt**	Within minutes - hours Within 24 hour Within a week- months	68 11 30	62.4 10.1 27.5
**Duration of preoccupation**	Minutes – hours in a day More than hours but within 24 hour in a day More than a day but within a week- months	79 22 8	72.5 20.2 7.3
**Preparedness**	No I didn’t have Chosen from what was available there Yes I have	43 52 14	39.4 47.7 12.8
**Reason for method used**	Because it was available at that time I thought this method may helpful I was sure that this method is suitable for me	46 41 22	42.2 37.6 20.2
**Anticipated consequences**	I proved to be failed in life, Worse for near and dear Might have affected in some way Nothing	51 35 23	46.8 32.1 21.1
**Responsibility for the attempt**	Provoked from outside Both me and external factor I am fully responsible	28 43 38	25.7 39.4 34.9
**Satisfaction regarding act before attempt**	Not at all To some extent Yes I was	49 52 8	45.0 47.7 7.3
**Resistance**	With much difficulty but finally decided Hesitating but finally decided No I didn’t	15 43 51	13.8 39.4 46.8
**Psycho-physiological state**	Very tensed up Little tensed up mostly relaxed Yes I was relaxed	28 57 24	25.7 52.3 22.0
**Emotional state during attempt**	Angry, blank Sadness Happiness	47 59 3	43.1 54.1 2.8
**Emotional control during attempt**	I was under control of emotion and thought Some control over emotion and thought Emotion and thought were under my control	53 44 12	48.6 40.4 11.0
**Any distraction during attempt**	Thinking many things but concentrate to do it Some thought about other thing No, I did not.	19 42 48	17.4 38.5 44.0
**Concern for anything during attempt**	About near / dear/other thing About some incomplete work /people Didn’t feel so	22 36 51	20.2 33.0 46.8
**Opinion about an attempt**	Try to overcome through different way. Be calm and think before any decision Should not do Should go for it once decided	59 48 2	54.1 44.0 1.8

**Table2:** Correlation of Process of Suicidal Attempt

**Demographic and Clinical Correlates of Process of Suicide**	
**Age **	Source of first information (0.282**), Emotional state during attempt (198*)
**Gender **	Any distraction during attempt (-0.190*)
**Marital status**	Duration of preoccupation (0.235*)
**SES**	Source of first information (-0.212*), Reason for method used ( 0.268**)
**Education **	Source of first information (-0.311**)
**Method **	Source of first information (0.216*)
**Any psychiatric diagnosis**	Duration since first death wish (-0.297**), Duration of decision to attempt (0.287**), Duration of preoccupation (0.307**), Preparedness (0.316**), Reason for method used (0.199*), Emotional state during attempt (0.324**)
**Family history of suicide**	Duration of first information (0.261**), Preparedness (0.220*).
**PSIS score**	Duration since first death wish (-0.508**), Duration of decision to attempt (0.566**), Duration of preoccupation( 0.300*),Preparedness ( 0.570**),Reason for method used ( 0.548**), Emotional state during attempt (0.382**), Psycho-physiological state ( 0.372**) Anticipated consequences ( 0.480**)

* P Value significant at the level of 0.05

** P Value significant at the level of 0.01

**Table3:** Scores of Demographic and Process of Suicide Attempt for the Perception of Suicide ‎Prevention

	**Sum of Squares**	**df**	**Mean Square**	**F**	**Sig.**
**Marital status**	0.718	1	0.718	5.633	0.019
**Occupation**	0.718	1	0.718	5.633	0.019
**Medical diagnosis**	1.033	1	1.033	8.303	0.005
**Prevention of others suicide**	7.844	1	7.844	129.017	0.000
**Duration since decision to attempt**	0.990	2	0.495	3.927	0.023
**Emotional control during attempt**	1.528	2	0.764	6.316	0.003
**Opinion about an attempt**	2.673	2	1.336	12.132	0.000

* P Value significant at the level of 0.05

** P Value significant at the level of 0.0

## Discussion

This study was aimed to find the relationship between perception of suicide prevention and ‎process of suicide attempt. As the treatment of psychiatric disorder, presence of psychotic ‎symptoms and severe physical illness or terminal illness may influence the process of ‎suicide attempt, such topics were excluded from this study. History of impairment in ‎cognitive function and ICD 10 diagnosis of dementia or mental retardation were excluded ‎due to issues related to reliability.‎

Process of Suicide Attempt

Most participants received the first information about suicide years back through the media. ‎This may be because mass media is easily accessible and available to the general ‎population. In general, people are more dependent on media than their own network for ‎social information due to the changing lifestyle (9). Media can influence the emotions, ‎behavior and psychological responses of the recipient and can change the recipients’ view of ‎the world by priming, agenda-setting, framing, cultivation and disinhibitory effect (10). ‎Social issues leading to suicide or suicide attempt have often been glorified or justified by ‎Indian media, encouraging the vulnerable people to identify themselves with the deceased ‎‎(11).‎

In this study, the majority of suicide attempts were impulsive, and characterized by the short ‎duration of decision to attempt and preoccupation, unpreparedness, sadness during the ‎attempt, and being in control of the emotions. Impulsivity is common in Asian population ‎and is associated with impulsive suicide or suicide attempt (12). As the majority had ‎experienced sadness before an attempt, they were predisposed to impulsive behavior. ‎Emotional exacerbation and emotional dysregulation are common before an attempt that ‎may interfere with rational decision-making and increase the focus on the immediate ‎interest (13).‎

We observed that the majority of the participants were unsatisfied with the act of attempt, ‎little sad and tried to resist the attempt. This indicates an ambiguous state of mind during ‎an attempt. Impulsive decisions are guided by intuition, but rationality and morality may ‎remain operative at the sub-threshold level, creating a conflict during decision- making (14).‎

Correlation of Suicide Process and Demographic Variables ‎

Females had more distractions during the attempt in this study. Gender differences exist in ‎taking responsibility and decision- making (15). It is often shaped by socio-cultural factors ‎such as norms, family, social status, beliefs and experiences (16). Women are more likely to ‎give value to familial and social responsibility before their personal preferences in life, and ‎this has reflected as distraction during an attempt. ‎

Because of easy access to media, younger and single participants appeared to access suicide ‎information from the media in this study. Middle and Low economic status is a risk factor ‎for impulsive suicide attempt, and the participants tended to use whatever method available ‎at the time of the attempt (17). Correlation of marital status and preoccupation revealed a ‎difference in the determinants of emotional processing such as support system and coping ‎and social learning (18).‎

Correlation of Suicide Process and Clinical Variables ‎

A positive correlation was found between the method used and the first source of ‎information about suicide. Poisoning has been the common method of suicide in India and ‎usually well covered in Indian media; this observation was likely as the media was the first ‎source for the majority of the participants. Psychiatric diagnosis had a significant correlation ‎with many variables of suicide process. This explains underlying mechanism such as the ‎stress diathesis of suicidality (19). Abnormality in serotonin functions and noradrenergic ‎neurotransmitter has been reported in suicide victims (20). Suicide and mental illness often ‎co-occur (17). Most psychiatric disorders have dysfunction of these neurotransmitters and ‎are concerned with impulsivity, emotional disturbances and error in decision- making. This ‎may explain our observation of a negative correlation with duration since the first death ‎wish and a positive correlation with duration of decision to attempt, duration of ‎preoccupation, preparedness and reason for the method used and emotional state during ‎attempt. Longer duration since the first information and more preparedness for an attempt ‎in those with a family history of suicide was due to acquired knowledge of completed suicide ‎by a family member (21). Consistent with previous reports, our analysis revealed a negative ‎correlation of PSIS score with duration since the first death wish: the higher the score in PSIS ‎the longer the duration. This may indicate persisting thoughts of suicide even after an ‎attempt suggestive of underlying a morbid psychological state. ‎

Severity of suicide intension appeared to be associated with a pattern of suicide process. ‎When the intention is stronger, the decision to attempt tends to be faster. The reason for ‎suicide attempt could be impulsivity or cognitive distortion such as dichotomous thinking, ‎cognitive rigidity, faulty logic or judgments that maintained suicidal preoccupation (22). ‎Emotion has an important role in the development of such cognition. According to ‎Baumeister’s paradoxical assumption, self-destructive behaviour is to protect oneself from ‎emotional distress (23). A state of negative urgency may result in an action without ‎preparation or anticipated consequences (24). In general, the positive correlation of many ‎variables of suicide process with the score on Pierce Suicide Intent Scale may validate other ‎observations in this study.‎

The Relationship between the Process of Suicide Attempt and Perception of Suicide ‎Prevention

As reported in previous studies, marital status has an effect on the prevalence and ‎perception of suicide prevention. In Asian countries, arranged marriage is most prevalent, in ‎which decisions are made by the family members. Forced or unwanted marriage, social ‎obligation, the strong association of family reputation with continuation of marriage, stigma ‎and social disapproval of divorce, reduced level of conflict tolerance and impulsivity leading ‎to an adjustment difficulty or family problem that lead to suicide is common in India (25, ‎‎^26^).‎

Group difference on the score of perception about suicide prevention for employment status ‎indicates the proximity of occupation related precipitating or perpetuating factors. In this ‎study, the majority were students or ‎homemakers. Increased incidence of suicide or suicide ‎attempt among students has been reported due to inability ‎to meet the high expectations, ‎love failure and financial problems (27). Homemakers are more likely to face ‎psychological ‎abuse, domestic violence, poor support from husbands and culture related ‎psychosocial ‎disadvantages.‎

Suicidal attempt associated with any medical illness depends on distress, associated ‎disability and expected ‎outcome (28). In our study, severe pain symptom was commonly ‎associated with medical condition. Patients with ‎pain often develop distress, hopelessness ‎and helplessness that may mediate suicidal behaviour. ‎

Similar to another report, we observed that a shorter duration of decision to attempt was ‎associated with elevated ‎perception that suicide can be prevented (29). This may indicate that ‎the decision was impulsive, without giving any ‎alternative or adequate thought. Baumeister ‎‎ (1990) proposed that on the experience of aversive self-awareness, a ‎person may develop a ‎state of cognitive deconstruction (constricted, present focused time perspective ‎and ‎cognitive rigidity) that leads to disinhibition resulting in non-resistance to impulsive suicide ‎attempt (30).‎

The level of emotional regulation may mediate a suicidal attempt in a predisposed person. ‎Emotional distress may ‎overpower the ability to make rational decisions based on external ‎stimulus (negative urgency), resulting in ‎maladaptive behavior among the attempters (31). ‎Intense emotions also result in a mood congruent memory biases, ‎thinking patterns and ‎evaluation of life that can influence behavior (32). After an attempt, the majority have ‎opined ‎against a suicide attempt. This further supports the role of emotion and mood during an ‎attempt. This may ‎reflect the different mental state after an attempt that may have ‎influenced the opinion about prevention of suicide ‎as reported in the community study (33).‎

## Limitations

One limitation of this study was its small sample size; thus, the relationships among ‎variables should be interpreted ‎with caution. As the study was conducted at a tertiary ‎centre, results are applicable to those availing in such centres. ‎Distinct psychological state ‎at the time of data collection may not necessarily be the same as ‎

the time of attempt. ‎Therefore, it may not represent the perception of prevention at the time of the attempt. Understanding the relationship between the process of suicide attempt and ‎perception of prevention may help to individualise the treatment and develop preventive ‎strategies.‎

## Conclusion

Our hypothesis was partially true. The perception of suicide prevention varies with some ‎variables of process of suicide attempt (duration since the decision to attempt and ‎emotional control during the attempt) and demographic characteristics (marital status, ‎occupation).
